# A Successfully Treated Case of Pediatric Traumatic Gastric Rupture Caused by Seat Belt Injury

**DOI:** 10.70352/scrj.cr.25-0547

**Published:** 2026-02-13

**Authors:** Yumiko Tabata, Shun Onishi, Nanako Nishida, Chihiro Kedoin, Ayaka Nagano, Yudai Tsuruno, Masakazu Murakami, Koshiro Sugita, Keisuke Yano, Takafumi Kawano, Satoshi Ieiri

**Affiliations:** 1 Department of Pediatric Surgery, Research Field in Medical and Health Sciences, Medical and Dental Area, Research and Education Assembly, Kagoshima University, Kagoshima, Kagoshima, Japan; 2 Division of Pediatric Surgery, Department of Surgery, Kobe University Graduate School of Medicine, Kobe, Hyogo, Japan; 3 Department of Pediatric Surgery, Hokkaido Medical Center for Child Health and Rehabilitation, Sapporo, Hokkaido, Japan

**Keywords:** pediatric surgery, complete gastric rupture, gastric rupture, traffic accident, seatbelt injury, blunt abdominal trauma, stomach injury

## Abstract

**INTRODUCTION:**

Gastrointestinal tract injury is estimated to occur in 0.9%–1% of blunt trauma cases, with gastric rupture occurring in approximately 3% of these cases. Blunt trauma injuries of the stomach are rare. In comparison to solid organ injuries, gastrointestinal tract injuries are less likely to be accompanied by hemorrhaging that can cause hemorrhagic shock, and the process is considered to be relatively slow.

**CASE PRESENTATION:**

The patient was a 9-year-old girl who was involved in a passenger-seat traffic accident and transferred to the emergency room of her previous hospital. She was then referred to our institution for further evaluation and treatment after active bleeding from the gastroepiploic artery, and a suspected duodenal injury was detected on contrast-enhanced CT. Upon arrival at our hospital, her vital signs were stable, and an angiogram showed no vascular bleeding. However, her blood examination results worsened; therefore, a contrast-enhanced CT scan was performed again 44 h after the injury. A definitive perforation on the anterior wall of the stomach was noted, and injuries to the liver and spleen were suspected. Emergency laparotomy was then performed. The abdominal cavity was filled with a large amount of bilious and serous ascitic fluid. Complete transection of the stomach was identified just proximal to the pylorus in the antrum. Based on contrast-enhanced CT findings showing that the gastroduodenal artery was intact, the blood supply to the stomach stump seemed to be preserved. Therefore, we decided to perform a gastric anastomosis without gastrectomy. End-to-end anastomosis was performed. Grade I injury was also observed in the spleen, but no other organ injury was observed. The patient began eating on POD 14 and was discharged on POD 27.

**CONCLUSIONS:**

During initial treatment, it is important to bear in mind that imaging studies may not always detect gastrointestinal tract injuries. Therefore, appropriate measures should be taken as necessary during subsequent treatment.

## INTRODUCTION

Gastric injuries are rarely observed after blunt abdominal trauma. It is estimated that damage to the gastrointestinal tract occurs in approximately 0.9%–1% of blunt trauma cases, with gastric rupture occurring in approximately 3% of these cases. Among all cases of blunt trauma, 0.025%–0.04% are associated with gastric rupture, indicating that blunt trauma rarely results in this injury.^1,2)^ Furthermore, injuries to the gastrointestinal tract are less likely to cause hemorrhagic shock than injuries to solid organs, and the process is considered relatively slow.

We herein report the case of a pediatric patient with gastric rupture caused by seatbelt trauma.

## CASE PRESENTATION

**Fig. 1:**
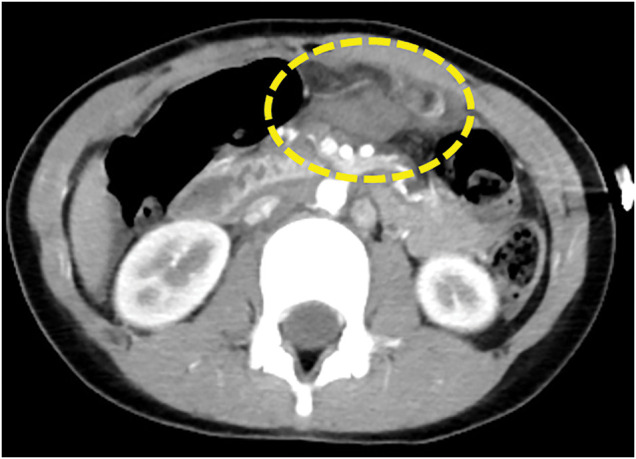
Contrast-enhanced CT 2 h after injury. Duodenal injury was suspected in the area marked by the yellow dotted line.

**Fig. 2:**
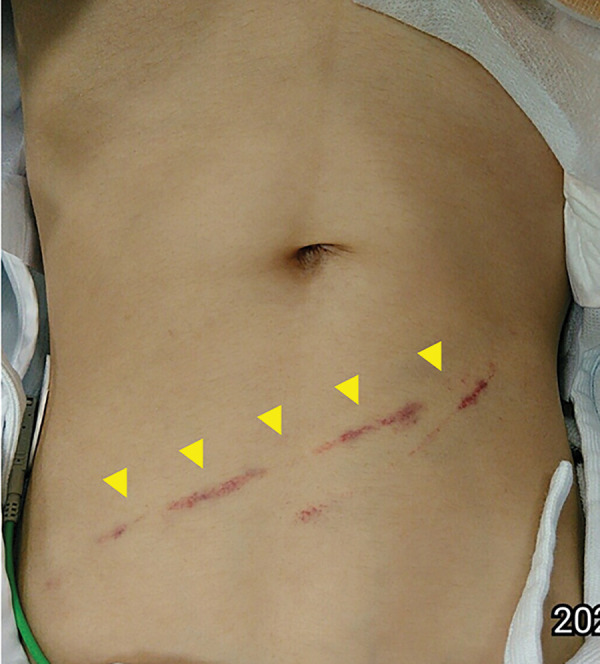
Seatbelt sign on the abdomen. The arrowheads indicate the seatbelt sign on the abdomen.

A 9-year-old girl was involved in a passenger-seat traffic accident and transferred to the emergency room of her previous hospital. She was referred to our department for further evaluation and management after contrast-enhanced CT suggested possible bleeding from the gastroepiploic artery and a duodenal injury (**Fig.1**). When she was transferred to our hospital, she was alert but complained of epigastric pain; seatbelt marks were noted on her abdomen (**Fig. 2**).

**Fig. 3:**
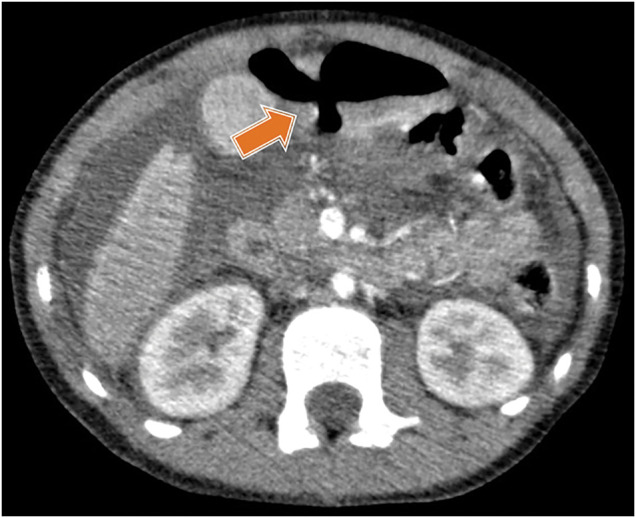
Contrast-enhanced CT 44 h after injury. Marked ascites and free air with an area of suspected injury were observed in the gastropyloric region region, which is indicated by the arrow.

The patient underwent emergent angiography under general anesthesia, but no active extravasation was observed. She was treated conservatively under sedation in the ICU. However, blood examinations performed the day after the injury and the following day revealed elevated amylase levels and a marked increase in C-reactive protein. The first contrast-enhanced CT scan performed 2 h after injury revealed no obvious free air or ascites. However, a follow-up second contrast-enhanced CT scan at 44 h after injury demonstrated significant free air and ascites in the abdominal cavity. A definitive perforation on the anterior wall of the stomach was noted, and injuries to the liver and spleen were suspected (**Fig. 3**).

**Fig. 4:**
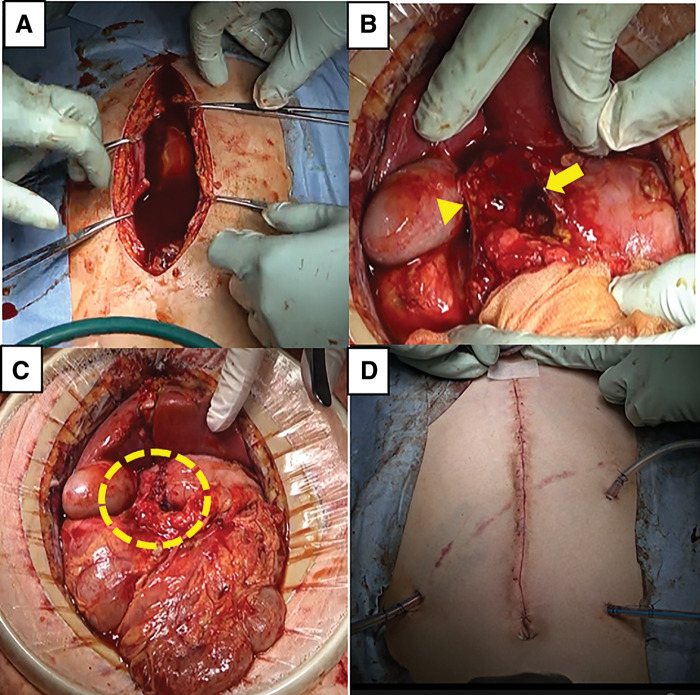
Operative findings. (**A**) Surgery was performed by making a 15-cm incision along the midline of the upper abdomen. A large amount of bile-like serous ascites was found in the abdominal cavity. (**B**) The stomach was amputated at the proximal mouth side of the pylorus (arrow, proximal gastric margin; arrowhead, distal gastric margin). The pyloric wall margins were intact. (**C**) Since the resection margins were intact and angiography at the time of admission confirmed preserved blood flow in the gastroduodenal artery, end-to-end anastomosis was deemed feasible. Posterior wall anastomosis was performed using the Albert technique with 4-0 PDS, and anterior wall anastomosis was performed using the Albert–Lembert technique. The anastomotic site is marked by the yellow dotted line. (**D**) Postoperative wound. Three drains were placed near the anastomotic site, at the foramen of Winslow, and in the Douglas pouch.

An emergency laparotomy was performed. The abdominal cavity was filled with a large amount of bilious and serous ascitic fluid (**Fig. 4A**). Complete transection of the stomach was identified just proximal to the pylorus in the antrum (**Fig. 4B**). Regarding organs other than the stomach, no obvious injuries were observed except for a subcapsular hematoma of approximately 1 cm at the splenic hilum, and there were no discolorations suggestive of tissue damage. Based on contrast-enhanced CT findings showing that the gastroduodenal artery was intact, the blood supply to the stomach stump seemed to be preserved. Therefore, we decided to perform a gastric anastomosis without gastrectomy. End-to-end anastomosis was performed using 4-0 absorbable monofilament sutures (PDS Plus; Johnson & Johnson, New Brunswick, NJ, USA) (**Fig. 4C**). Three drains were placed near the anastomotic site, at the foramen of Winslow, and in the pouch of Douglas (**Fig. 4D**). A nasogastric tube was placed for gastric decompression, and a nasojejunal tube was inserted as a trans-anastomotic tube; the operation was then completed.

Since *Candida albicans* was detected in the ascitic fluid collected intraoperatively, treatment with tazobactam/piperacillin and micafungin was initiated on POD 2. Contrast-enhanced CT performed on POD 6 revealed a subdiaphragmatic abscess. Neither addition nor modification of antibiotics was made, and no interventions such as drainage were performed for the subdiaphragmatic abscess. The patient's vital signs were stable, and she was transferred from the ICU to the pediatric ward on POD 7. A contrast study of the upper gastrointestinal tract on POD 14 revealed no evidence of anastomotic leakage; therefore, oral feeding was initiated. Contrast-enhanced CT on POD 26 showed improvement in the subdiaphragmatic abscess, and the patient was discharged. Endoscopic examination performed 5 months after surgery showed no anastomotic stenosis or other abnormalities.

## DISCUSSION

Injuries to the gastrointestinal tract due to blunt trauma are reported to account for approximately 1% of all traumatic injuries, with 0.8% of all injuries involving the jejunum/ileum, 0.3% involving the colon/rectum, 0.1% involving the duodenum, and 0.04% involving the stomach, with some overlap.^1)^ It has been demonstrated that gastric rupture is a rare injury, accounting for 0.02%–1.7% of all blunt trauma injuries.^3–7)^ The most frequent cause of injury is traffic-related trauma, which includes blunt abdominal trauma from steering wheels and seatbelt injury. The reasons for the rarity of gastric injuries due to blunt trauma include the location of the stomach in the epigastrium, its protection by the thorax and liver, the thick and resilient stomach wall, its relatively loose fixation and mobility, and its large luminal capacity. As a result, gastric injuries are frequently associated with high-energy trauma, a condition that often results in accompanying damage to other intra-abdominal organs.

Previous studies have reported that gastric rupture is more common in younger patients and when the stomach is distended with food.^5)^ The anterior wall and the greater curvature are the most frequently affected sites. The proposed mechanisms include a sudden rise in intragastric pressure caused by external compression or rapid deceleration, leading to tearing of the gastric wall, particularly along the greater curvature or the anterior wall.^3,8)^ Compression of the stomach between the abdominal wall and the vertebral column can further contribute to injury and is often accompanied by damage to adjacent organs, such as the spleen or pancreas. In Japan, gastric rupture typically involves the body of the stomach, whereas complete gastric transection tends to occur in the antrum.

In the present case, it was not possible to confirm whether the patient had a full stomach at the time of the traffic accident, as her pre-injury intake could not be confirmed. However, rupture occurred in the anterior region, consistent with previous reports. Grade I splenic injury was also identified as a coexisting complication, but no other significant injuries were noted. The most likely mechanism of gastric rupture was a sudden increase in pressure on the stomach due to deceleration forces, as the injury was located in the gastric antrum region, and the vehicle in which the patient was riding came to a sudden stop in a traffic accident. In addition, the presence of seatbelt marks suggests that the seatbelt may have affected the external pressure on the abdomen. The combined effects of compression between the seatbelt and lumbar spine may have contributed to gastric rupture. Therefore, both factors are considered potential contributing factors in the present case.

Since seatbelts are primarily designed for adults, proper positioning across the pelvis and shoulder can be difficult in children, leading to slippage or malalignment.^9)^ As a result, correct placement of the belt over the anterior superior iliac spine and shoulder is often challenging in pediatric occupants. Nevertheless, when correctly positioned, seatbelts significantly reduce the risk of injury and mortality in motor vehicle collisions.^10)^ In contrast, improperly fastened seatbelts provide limited protective effect and may even increase the risk of intra-abdominal injury, as the belt can ride up onto the abdomen and exert direct compressive force on the stomach or other hollow organs.^11,12)^ In the present case, the seatbelt was not properly fastened, and a seatbelt mark was observed extending from the right anterior superior iliac spine to the left subcostal region. The mark traversed the upper abdomen, suggesting that the seatbelt had pressed deeply into the abdomen at the time of the collision, directly compressing and injuring the stomach. Hollow viscus injuries are often difficult to detect on initial imaging and may only become apparent after clinical deterioration. However, previous studies have reported that more than 65%–80% of patients presenting with a seatbelt sign have associated hollow viscus injuries.^12,13)^ In this case, the presence and location of the seatbelt mark indicated improper seatbelt use, and even in the absence of imaging abnormalities, hollow viscus injury should have been strongly suspected, warranting close and repeated imaging follow-up.

In cases of gastric rupture, primary repair by direct suture of the perforation site is generally considered the standard approach. In contrast, in Japan, complete gastric transection is often managed with gastrectomy followed by reconstruction. One of the main reasons for performing a gastrectomy is severe tissue destruction at the site of transection. In the present case, resection was deemed unnecessary, and end-to-end anastomosis was performed. This decision was based on several factors: the resection margins at the transection site were smooth and well-defined, preoperative angiography showed no injury to the gastric or gastroduodenal arteries, and the damage was confined to the stomach with no injuries to surrounding organs requiring repair. Although anastomotic leakage can significantly worsen postoperative outcomes and thus requires careful consideration, in this case, the risk was considered low. Moreover, performing an end-to-end anastomosis allowed preservation of the physiological continuity of the gastrointestinal tract, which was judged to outweigh potential risks.

Injuries to the gastrointestinal tract rarely result in hemorrhagic shock, and the post-injury course tends to be relatively stable in comparison to injuries to solid organs. Although gastrointestinal tract injuries are more likely to be detected when free air is recognized on radiography or CT, free air is often not present on imaging studies immediately after the injury. In cases of gastric rupture, the reliability of detecting free air on radiographic examination is low, with reports indicating that free air is absent in >50% of cases. In the present case, the absence of free air on CT imaging performed 2 h after injury was particularly notable.^1)^

On admission, the patient presented with tachycardia; however, her blood pressure was stable and she was alert. Initially, the injury was too small to be detected using imaging or other examinations. However, over time, the injury may have progressed, ultimately leading to rupture. Although the diagnosis of gastric rupture was delayed due to a lack of awareness at the initial presentation, appropriate diagnostic evaluations and treatment were subsequently performed based on the patient’s condition, resulting in successful preservation of the gastric function.

Most complications resulting from gastric rupture are associated with sepsis, and the reported mortality rate ranges from 0% to 66%.^1–4,14)^ The high mortality rate associated with gastric rupture is thought to be due to the anatomical complexity of the stomach, which makes it prone to severe injuries. Such injuries are often the result of high-energy trauma and are frequently accompanied by extensive damage to other organs. However, in the present case, the damage was limited to the stomach, with only mild Grade I injury to the spleen.

## CONCLUSIONS

Gastric rupture due to blunt trauma is rare and cases occurring without significant injury to the other organs are even rarer. It should be noted that gastrointestinal injuries may not be apparent on imaging studies at the initial presentation. Therefore, if such injuries are suspected, appropriate follow-up evaluations should be performed during the course of treatment. On the other hand, even if gastrointestinal injury is not diagnosed initially, careful monitoring and timely intervention can be life-saving. Moreover, even in cases of complete gastric transection, end-to-end anastomosis may be performed without gastrectomy when certain conditions are met.

## DECLARATIONS

### Availability of data and materials

The data used in this report are available from the corresponding author on request.
